# Cyclic thrombospondin-1 mimetics: grafting of a thrombospondin sequence into circular disulfide-rich frameworks to inhibit endothelial cell migration

**DOI:** 10.1042/BSR20150210

**Published:** 2015-11-26

**Authors:** Lai Yue Chan, David J. Craik, Norelle L. Daly

**Affiliations:** * The University of Queensland, Institute for Molecular Bioscience, Brisbane 4072, Queensland, Australia; † Centre for Biodiscovery and Molecular Development of Therapeutics, AITHM, James Cook University, Cairns 4870, Queensland, Australia

**Keywords:** angiogenesis inhibitors, endothelial cells, non-toxic, peptide, stable, thrombospondin-1

## Abstract

Tumour formation is dependent on nutrient and oxygen supply from adjacent blood vessels. Angiogenesis inhibitors can play a vital role in controlling blood vessel formation and consequently tumour progression by inhibiting endothelial cell proliferation, sprouting and migration. The primary aim of the present study was to design cyclic thrombospondin-1 (TSP-1) mimetics using disulfide-rich frameworks for anti-angiogenesis therapies and to determine whether these peptides have better potency than the linear parent peptide. A short anti-angiogenic heptapeptide fragment from TSP-1 (GVITRIR) was incorporated into two cyclic disulfide-rich frameworks, namely MCoTI-II (*Momordica cochinchinensis* trypsin inhibitor-II) and SFTI-1 (sunflower trypsin inhibitor-1). The cyclic peptides were chemically synthesized and folded in oxidation buffers, before being tested in a series of *in vitro* evaluations. Incorporation of the bioactive heptapeptide fragment into the cyclic frameworks resulted in peptides that inhibited microvascular endothelial cell migration, and had no toxicity against normal primary human endothelial cells or cancer cells. Importantly, all of the designed cyclic TSP-1 mimetics were far more stable than the linear heptapeptide in human serum. The present study has demonstrated a novel approach to stabilize the active region of TSP-1. The anti-angiogenic activity of the native TSP-1 active fragment was maintained in the new TSP-1 mimetics and the results provide a new chemical approach for the design of TSP-1 mimetics.

## Abstract (precis)

The findings suggest re-engineered cyclic TSP-1 mimetics are non-toxic, highly stable, and possess potent anti-angiogenesis activity without altering the native fold of the cyclic frameworks. This provides an alternative approach for cancer drug development particularly in the thrombospondin field.

## INTRODUCTION

Tumour formation is dependent on nutrient and oxygen supply from adjacent blood vessels [1]. An increase in new blood vessel formation during tumour angiogenesis will enhance tumour growth and provide an opportunity for cancer cells to metastasize [2]. In the event of tumour angiogenesis, a range of angiogenic growth factors is induced, including vascular endothelial growth factor (VEGF) [3], platelet-derived growth factor (PDGF) and basic fibroblast growth factor (bFGF) [4]. These angiogenic growth factors can increase proliferation, sprouting and migration in endothelial cells [5]. Endogenous angiogenesis inhibitors therefore play an important role in regulating tumour formation [6].

Thrombospondin-1 (TSP-1) [7], a naturally occurring angiogenesis inhibitor, has been studied extensively due to its potent anti-angiogenic activity observed in a range of *in vitro* and *in vivo* experiments [8]. This 450-kDa protein with multiple domains [9] has a broad range of functions, including modulation of platelet aggregation, inflammation and angiogenesis [10,11]. Only specific regions of TSP-1 contain sequences that inhibit angiogenesis, including a seven residue fragment (GVITRIR) encompassing residues 454–460 of the human TSP-1 protein (Figure 1A), which has been shown to inhibit migration of endothelial cells via interaction with CD36 [12–14]. CD36 is a scavenger receptor located on the cell surface and expressed in a broad range of mammalian cells (normal and disease tissues) including microvascular endothelial cells, phagocytes, adipocytes and hematopoietic cells [15]. Given its diverse expression pattern on different cell types, it has been associated with multiple biological functions via mediating cell-specific responses [16,17]. Among all biological functions exhibited by CD36, the CD36-TSP-1 interaction is known to be an important process that acts as a negative regulator of angiogenesis [17].

**Figure 1:**
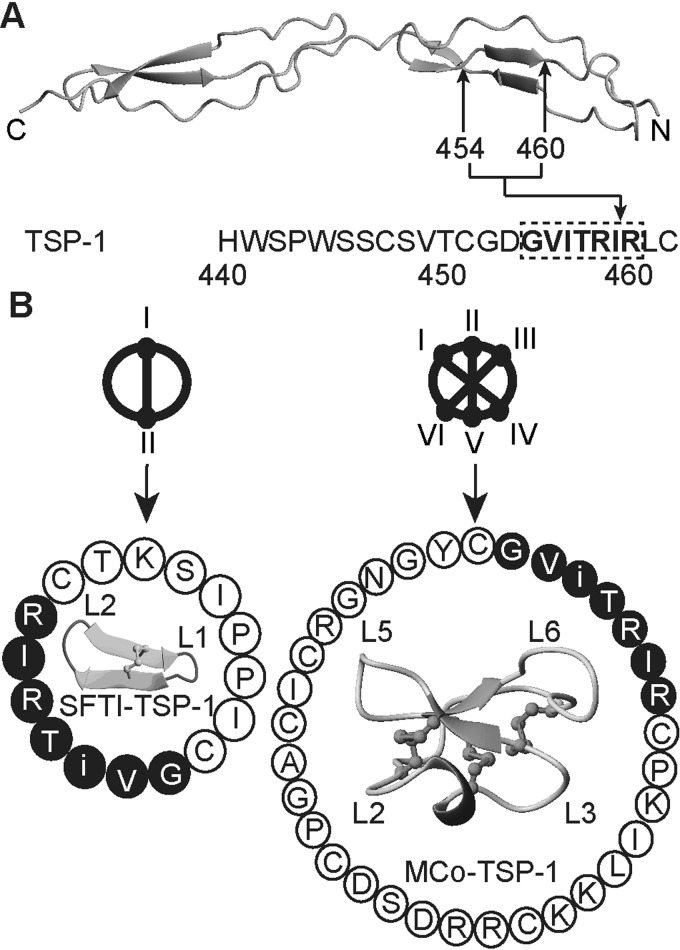
Structure of human thrombospondin (TSP-1) and a schematic representation of the approach used for the design of cyclic TSP-1 mimetics (**A**) The crystal structure of the human thrombospondin (TSP-1) type 1 repeat domains 2 and 3 was obtained from the protein data bank (www.pdb.org; PDB ID: 3R6B). The ribbon diagram of the structure was displayed using the MolMol program [61] and the human TSP-1 sequence was obtained from Uniprot (Accession No. P07996). The human TSP-1 region corresponding to residues 454–460 (GVITRIR), highlighted using dashed lines, was previously shown to confer broad-spectrum anti-angiogenic activity and therefore was selected for the present study [9]. (**B**) This active region (marked by black amino acid residues) was incorporated into two cyclic disulfide-rich frameworks, i.e., SFTI-1 (PDB ID: 1JBL) and MCoTI-II (PDB ID: 1HA9) (three-dimensional structures shown inside circular amino acid sequence). The disulfide bond connectivity is illustrated schematically (upper half of panel), with cysteines labelled using Roman numerals. The first isoleucine of the heptapeptide was modified to a D-Ile, as previous studies suggested its bioactivity could be enhanced. Different loops of the cyclic framework are represented as ‘L1, L2 etc.’

Various chemical approaches, including truncations of the original anti-angiogenic region of TSP-1 and unnatural amino acid modifications, have been employed to modify the active heptapeptide fragment to achieve better inhibition of angiogenesis [13,14]. An example of a synthetic peptide derived from TSP-1 is the nonapeptide ABT-510, which reached phase II clinical trials for the treatment of metastatic melanoma but these trials were terminated due to a lack of clinical efficacy [18]. It has been suggested that a higher dosage or combination with other cytotoxic drugs could improve the probability of ABT-510 becoming a successful drug [18]. However, such approaches could increase the risk of toxicity or adverse side effects and are likely to be more costly to patients.

Another approach, designed to overcome these potential risks, involves grafting the active heptapeptide into cyclic disulfide-rich peptides [19], which are reported to have enhanced stability over linear peptides [20]. In general, peptides have a range of advantages over small-molecule drugs, including lower toxicity, high potency, selectivity and an ability to target a broad range of diseases [21]. The naturally occurring cyclic disulfide-rich peptides used in the present study are a 34-residue *Momordica cochinchinensis* trypsin inhibitor-II (MCoTI-II) [22] and a 14-residue sunflower trypsin inhibitor-1 (SFTI-1) [23] (Figure 1B). Although these frameworks naturally have trypsin inhibitory activity, this activity can be abolished by a point mutation of the active site residue [24]. Both frameworks have exceptional stability, are non-toxic, and have the ability to resist thermal or enzymatic degradation. The stability of these frameworks is due to the cyclic cystine knot (CCK) motif of MCoTI-II [19,25], and the extensive hydrogen bonding network of SFTI-1 [26]. Cyclic disulfide-rich peptides can be readily re-engineered due to the availability of multiple loops within their frameworks, with the possibility to incorporate a foreign active sequence into these loops for delivery to a specific receptor. This approach has been used successfully in more than a dozen studies that yielded peptides with good potency towards targets of interest [27–32].

The present study examines the prospect of grafting the heptapeptide TSP-1 fragment into specific loops of MCoTI-II and SFTI-1 to design a new suite of cyclic TSP-1 mimetics with the ability to inhibit endothelial cell migration. The anti-angiogenic heptapeptide from TSP-1 was chosen based on its ability to inhibit migration of endothelial cells, and the goal was to use cyclization to improve the potency and stability of the TSP-1 fragment without introducing toxicity. The rationale for the present study is illustrated in Figure 1(B). The newly designed cyclic TSP-1 mimetics were chemically synthesized using native chemical ligation [33] and were screened in a range of *in vitro* assays. Grafting an active TSP-1 fragment into cyclic disulfide-rich frameworks is a novel approach for the TSP-1 research area and in the current study we have produced promising peptide-based inhibitors for targeting a range of blood vessel overgrowth diseases such as tumour angiogenesis, atherosclerosis and retinal disease.

## EXPERIMENTAL

### Peptide synthesis

The heptapeptide TSP-1 (GViTRIR), where ‘i’ is D-isoleucine, was grafted into two cyclic disulfide-rich frameworks (MCoTI-II and SFTI-1) to form MCo-TSP-1 and SFTI-TSP-1. Both grafted peptides were synthesized using manual solid-phase peptide synthesis on a CS Bio synthesizer using Boc chemistry. In this method, Boc-Gly-PAM resin was used along with *S*-tritylmercaptopropionic acid as a linker, and using *N,N,N′,N′*-tetramethyl-*O*-(6-chloro-1H-benzotriazol-1-yl)uronium hexafluorophosphate (HCTU) for amino acid activation. The linear heptapeptide was synthesized on a Symphony microwave synthesizer using chlorotritylchloride resin and with Fmoc chemistry using piperidine for amino acid activation. The linear heptapeptide was constructed with normal N- and C-termini without chemical modifications. The peptides were synthesized on a 0.5 mmol scale. Peptides made using Boc chemistry were cleaved from the resin using hydrogen fluoride with *p*-cresol as a scavenger at 0 to 5°C for 1 h. Fmoc-synthesized peptides were cleaved using a mixture of 95% (v/v) trifluoroacetic acid/2.5% (v/v) triisopropylsilane/2.5% (v/v) H_2_O. Traces of trifluoroacetic acid were removed using a rotary evaporator, and the remaining peptide in solution was precipitated with diethyl ether and extracted in 50% (v/v) acetonitrile/50% (v/v) H_2_O. The aqueous layer was lyophilized and the resulting crude peptides were purified using Shimadzu reverse-phase HPLC. Native SFTI-1 was synthesized using Boc chemistry and MCoTI-II was isolated directly from *M. cochinchinensis* seed extract as described by Chan et al. [34].

### Peptide purification and folding conditions

Crude peptides obtained from either plant extracts or chemical synthesis were purified using a series of Phenomenex C18 columns on reverse-phase HPLC. Gradients of 1%/min of 0–80% solvent B (90% (v/v) acetonitrile in 0.045% (v/v) trifluoroacetic acid in H_2_O) and solvent A (0.05% (v/v) trifluoroacetic acid in H_2_O) were used and the eluent was monitored at 215 and 280 nm. The purity of the peptides was examined on a Nexera ultra HPLC (Shimadzu) with a flow rate of 0.4 mL/min on a 0.8 mL/min Agilent column using a 2% gradient of 0–50% solvent B and masses were determined by electrospray mass spectrometry. Only peptides with greater than 95% purity were used in assays. MCo-TSP-1 peptide was folded in one-pot oxidation solution, which contained 0.1 M ammonium bicarbonate (pH 8.5) for 24 h. Both native SFTI-1 and SFTI-TSP-1 peptides were folded in a two-step oxidation condition, which included cyclization of the peptide with 0.1 M ammonium bicarbonate (pH 8.0)/0.1 M TCEP for 24 h then oxidized in 0.1 M ammonium bicarbonate (pH 8.0) for another 24 h. MCoTI-II did not need to be oxidized, as it was isolated directly from the seed extract.

### Cell culture

HUVECs were cultured in EGM (endothelial cell growth media)-2 BulletKit supplemented with SingleQuots (endothelial basal media-2 (EBM-2) supplemented with growth factors, cytokines, antibiotics; Lonza)/10% (v/v) FBS). HMVECs were cultured in EGM-MV BulletKit supplemented with SingleQuots (EBM-2 supplemented with growth factors, cytokines, antibiotics; Lonza)/20% (v/v) FBS. HT-29 (colon cancer cells) and MCF-7 cells (breast cancer cells) were cultured in 10% (v/v) FBS/DMEM with 1% (v/v) penicillin–streptomycin (5000 U/mL; Life Technologies). For PC3 cells (prostate cancer cells), 10% (v/v) FBS/RPMI 1640 media with 1% (v/v) penicillin–streptomycin (5000 U/mL; Life Technologies) was used during culture. All cells were maintained at 37°C in 5% CO_2_.

### Cell cytotoxicity assay

All cells were maintained using the media conditions described above, and passages 2–10 were used for all cell lines. 5×10^3^ cells/well (100 μL) were used for both HUVECs and HMVECs, and 2.5×10^3^ cells/well (100 μL) were used for other cell lines in this assay. All cells were allowed to attach for 24 h after plating. Cells were treated with fresh media the next day, before the addition of peptides. After the addition of peptides (at a range of final concentrations: 10 μL; 50 μM, 25 μM, 10 μM, 5 μM, 1 μM, 0.5 μM, 0.1 μM and 0.01 μM), cells were incubated for 2 h. Except for HMVECs, the final peptide concentration range tested was 10-fold dilutions from 10 μM to 0.00001 μM (10 μL) and these cells were incubated for 24 h. 0.1% (v/v) Triton X-100 (10 μL) was used as a positive control. After the 2-h incubation, 3-(4,5-dimethylthiazolyl-2)-2,5-diphenyltetrazolium bromide (MTT) (10 μL; 5 mg/mL in PBS) was added, and cells were incubated for a further 4 h. Supernatant was then removed and 100 μL DMSO added to solubilize formazan salts. Experiments were performed in triplicate. Cell numbers were followed at 600 nm.

### Cell proliferation assay

All cells were maintained using the media conditions described above, and passages 2–10 were used for all cell lines. Cell numbers of 2.5×10^3^ cells/well (100 μL) were used for both HUVECs and HMVECs, and 1.5×10^3^ cells/well (100 μL) were used for other cell lines in this assay. All cells were allowed to attach for 24 h after plating. Cells were treated with fresh media the next day, before the addition of peptides and then incubated for 72 h. Similar peptide concentrations to those mentioned above (in the cell cytotoxicity section) were used. 0.1% (v/v) Triton X-100 (10 μL) was used as a positive control. After 72 h of incubation, MTT and DMSO were added and analysed using the same method as described above.

### Transwell migration assay using HUVECs

An endothelial cell migration assay was conducted to determine the anti-angiogenic activity of newly designed cyclic TSP-1 mimetics. This assay was established based on previous studies using a HUVEC migration assay [35–38]. A 24-well plate loaded with 8 μm polycarbonate inserts (Corning) was used. Prior to the assay, all inserts were pre-coated with attachment factor (Gibco) for 30 min. Passages 6–10 were used for HUVECs, and were maintained in the conditions described above. Prior to the start of this assay, HUVECs were serum-starved overnight (16 h) and washed with PBS. HUVECs were detached with non-enzymatic cells dissociation buffer (Gibco) for 10 min, and resuspended in 0.1% (v/v) FBS/EBM-2 and centrifuged at 347 ***g*** for 8 min. Peptides in varying concentrations (0.001–50 μM) were added to the cell suspension and incubated for 30 min at 37°C before adding into the insert. HUVECs with a cell number of 1×10^6^ cells/well (100 μL) were seeded to the apical well and 600 μL media supplemented either with or without 0.3 nM VEGF was added to the basolateral well. Cells with 0.1% (v/v) FBS/EBM-2 basal media were used as negative controls, whereas cells with 0.3 nM of VEGF were used as positive controls. Cells were allowed to migrate for 4 h at 37°C. After incubation, the cell suspension from the top chamber was gently removed using a cotton swab and the insert was fixed with 4% (v/v) formaldehyde/PBS and stained with 0.05% (v/v) crystal violet/PBS. Next, cells were destained with 10% (v/v) acetic acid/H_2_O. For each insert, 100 μL of the destained solution was transferred into a 96-well plate and absorbance was read using Power Wave XS (Biotek) spectrometer at 590 nm. Experiments were done in triplicate.

### Transwell migration assay using HMVECs

This assay was established in a similar manner as described above; however, additional procedures specifically for HMVECs were included based on previous studies [39–41]. HMVECs within passages 7–12 were used and maintained as above. Prior to the start of the assay, HMVECs were serum-starved overnight (16 h) and washed with PBS. HMVECs were detached with 0.25% (w/v) trypsin/EDTA solution (Gibco) for 2 min, and resuspended in 0.1% (v/v) FBS/EBM-2 media only and centrifuged at 347 ***g*** for 2 min. Peptides at various concentrations (0.00001–10 μM) were added to the cell suspension and incubated for 30 min at 37°C before adding into the insert. HMVECs with a cell number of 0.5×10^6^ cells/well (100 μL) were seeded to the apical well and 600 μL of media supplemented with/without 1 nM VEGF/5% FBS was added to the basolateral well. Cells with 0.1% (v/v) FBS/EBM-2 basal media was used as a negative control, whereas cells with 1 nM of VEGF/5% (v/v) FBS were used as a positive control. Cells were allowed to migrate for 24 h at 37°C. After incubation, cells were treated as described above, for the HUVEC migration assay.

### Hemolytic assay

Human red blood cells were used to measure the hemolysis of all peptides, with melittin used as a positive control. Peptides concentrations were 50 μM, 25 μM, 10 μM, 5 μM, 1 μM, 0.5 μM, 0.1 μM and 0.01 μM. Melittin was tested at eight concentrations, with two-fold dilution from a concentration of 20 μM. Sample absorbance was measured at 415 nM using a BioTek PowerWave XS instrument, as described previously [29]. Experiments were done in triplicate.

### Stability assay

All peptides were tested on human serum from male AB plasma (Sigma–Aldrich) at an initial concentration of 300 μM. Peptide incubation time points were 0, 2, 4, 8, 16 and 24 h. Samples were prepared according to previously described methods [29]. Briefly, 90 μL of supernatant was taken at each time point and chromatographed on a 0.3 mL/min Phenomenex column using a linear 1%/min gradient of 0–50% solvent B on an analytical reverse-phase HPLC (Agilent). The elution times for all peptides were determined by the serum control at 0 h. The stability of each peptide for each time point was calculated as the height of the serum-treated peptide peak at 215 nm as percentage of the height of the 0-h serum-treated control. Experiments were done in triplicate.

### NMR analysis

Peptides were dissolved in 90% (v/v) H_2_O/10% (v/v) D_2_O at a concentration of 1 mM at pH 5.5, using D_2_O at 99.9% purity obtained from Cambridge Isotope Laboratories, Woburn, MA. One-dimensional (^1^H) and two-dimensional (TOCSY and NOESY) spectra were recorded at 298 K on a Bruker Avance 600 MHz spectrometer. Mixing times of 200–300 ms were employed for NOESY experiments. Spectra were assigned using CCPNMR [42].

### Statistical analysis

All statistical analyses were done using GraphPad Prism version 6. All data represent average mean ± S.D. Statistical significance were performed using one-way analysis of variance (ANOVA) with Dunnett's multiple comparison test was applied in the analysis. *P*≤0.05 was considered statistical significant.

## RESULTS

### Design and synthesis approach of cyclic TSP-1 mimetics

The sequences of the synthetic peptides are given in Table 1. The first isoleucine residue in the TSP-1 heptapeptide was modified to a D-amino acid due to the enhanced activity observed with this modification in a previous study [13]. The heptapeptide was incorporated into the secondary loop (loop 2) of SFTI-1 and loop 6 of MCoTI-II. Native chemical ligation and Boc chemistry methods were employed to cyclize the N- and C-termini of cyclic TSP-1 mimetics (MCo-TSP-1 and SFTI-TSP-1) [33], which were then oxidized to form the native disulfide bonds. Synthetic SFTI-1 was oxidized according to procedures outlined in the methods section, whereas MCoTI-II was isolated directly from a seed extract. Only peptides that were correctly folded, had the correct mass and were greater than 95% pure were used in the *in vitro* tests (Supplementary Figure S1). All peptides were readily soluble in water, which allowed them to be tested in a range of cell-based assays.

**Table 1:** Peptide sequences involved in the present study *Cyclic peptide. †The lower-case ‘i’ represents D-isoleucine (D-Ile).

Peptides	Sequences	Molecular mass (Da)
TSP-1	**GViTRIR**†	814.00
SFTI-1*	CTKSIPPICFPDGR	1513.79
SFTI-TSP-1*	CTKSIPPIC**GViTRIR**†	1737.15
MCoTI-II*	CPKILKKCRRDSDCPGACICRGNG-YCGSGSDGGV	3452.94
MCo-TSP-1*	CPKILKKCRRDSDCPGACICRGNG-YC**GViTRIR**†	3632.33

### NMR analysis of the re-engineered cyclic TSP-1 mimetics

It was important to analyse the structure of the newly designed cyclic TSP-1 analogues to determine whether these peptides adopt similar conformations to their native frameworks. In general, the αH NMR chemical shifts of MCo-TSP-1 and SFTI-TSP-1 were found to be similar to MCoTI-II and SFTI-1, respectively, which indicates that the native fold was maintained in the grafted peptides (Figure 2). There were some differences in αH NMR chemical shifts at the grafted regions, but this was expected due to the differences in amino acid residues in these regions.

**Figure 2:**
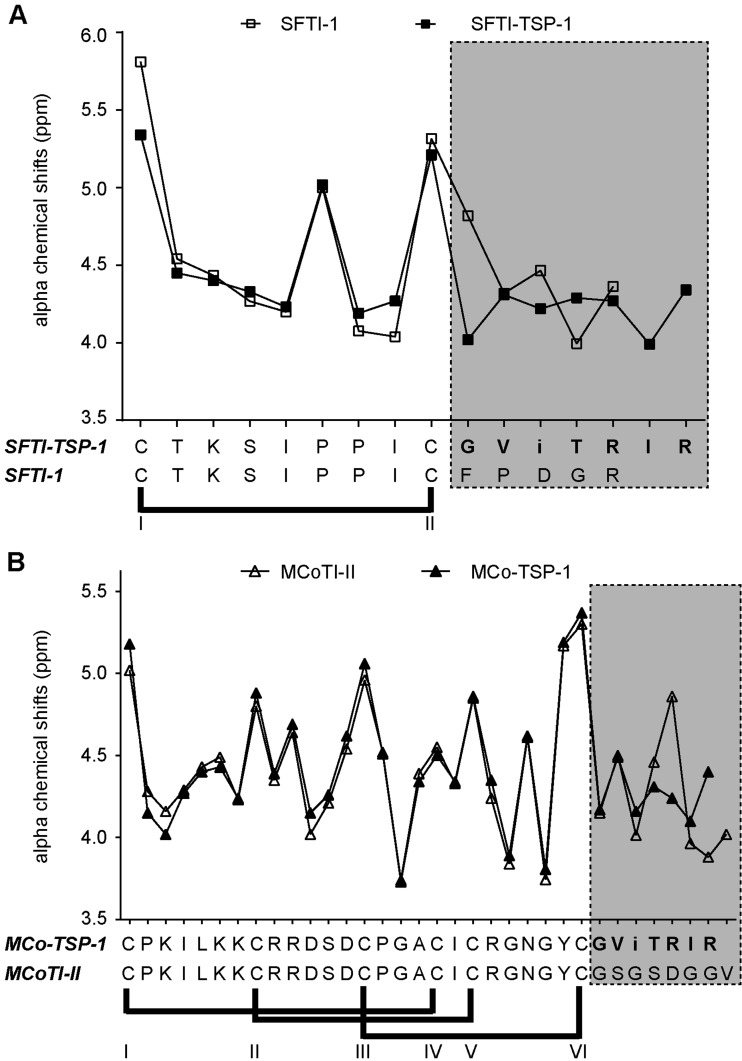
NMR analysis of cyclic TSP-1 mimetics (**A**) Comparison of SFTI-1 and SFTI-TSP-1 αH chemical shifts. (**B**) Comparison of MCoTI-II and MCo-TSP-1 αH chemical shifts. All 1H NMR spectra were recorded at 298 K. The active TSP-1 fragment is highlighted in bold and the region for comparing native and inserted sequence in different cyclic frameworks is in a grey box. Disulfide bond connectivity is indicated with bold lines and each cysteine is labelled with Roman numerals.

### Endothelial cell migration on HUVECs and HMVECs

We tested the inhibitory activity of our newly designed cyclic TSP-1 mimetics against both HMVECs and HUVECs, as previous studies reported that peptides derived from TSP-1 have the ability to inhibit endothelial cell migration more potently in HMVECs [8,12] (a cell line that overexpresses CD36) than HUVECs (a cell line that expresses integrins but not CD36) [35]. Based on the low expression levels of CD36 in cancer cells (PC-3, MCF-7 and HT-29) showed by CD36 antibody staining (PC-3 and MCF-7) from the Human Protein Atlas portal (www.proteinatlas.org) [43] and CD36 RNA expression of HT-29 and MCF-7 from the Expression Atlas (http://www.ebi.ac.uk/gxa) [44] databases, these cell lines were not examined in the cell migration assay. The inhibitory activity of the peptides was compared with VEGF, a potent growth factor that stimulates angiogenesis. We found that the TSP-1 heptapeptide, SFTI-TSP-1 and MCo-TSP-1 could more effectively suppress the migration of HMVECs than HUVECs (Figures 3A and 3B). Among the peptides tested on HMVECs, MCo-TSP-1 was more effective at suppressing migration compared with SFTI-TSP-1 and linear TSP-1 heptapeptide (Figure 3B). At 10 μM, the linear TSP-1 heptapeptide, SFTI-TSP-1 and MCo-TSP-1 displayed 20%, 45% and 30% higher inhibition of HMVECs, respectively, when compared with the same concentration of these peptides incubated with HUVECs. At 1 μM, SFTI-TSP-1 and MCo-TSP-1 displayed 29% and 41% higher inhibition of HMVEC migration, respectively, compared with HUVECs at the same concentration. Interestingly, MCo-TSP-1 still conferred approximately 20% inhibition on HMVEC migration at 100 nM. Both native frameworks did not have an inhibitory effect either on HUVECs (data not shown) or HMVECs (Figure 3C). Overall, the newly designed cyclic TSP-1 mimetics had enhanced inhibition of HUVEC or HMVEC migration compared with the linear TSP-1 heptapeptide.

**Figure 3:**
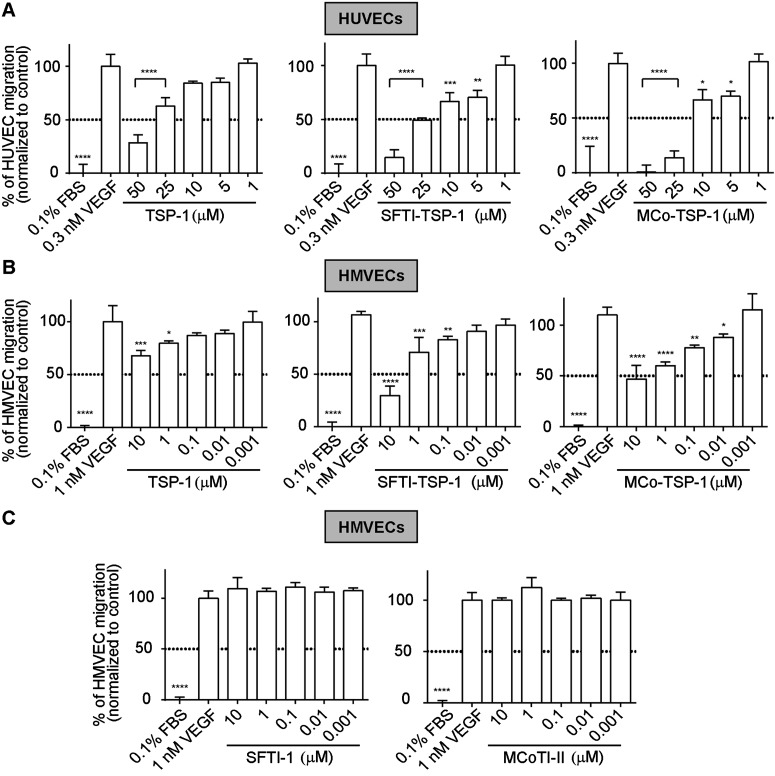
Endothelial cell migration assays for HUVECs and HMVECs (**A**) The percentage of HUVEC migration following treatment with TSP-1, SFTI-TSP-1 and MCo-TSP-1. (**B**) The percentage of HMVEC migration following treatment with TSP-1, SFTI-TSP-1 and MCo-TSP-1. (**C**) The percentage of HMVEC migration following treatment with native cyclic frameworks, SFTI-1 and MCoTI-II. All peptides were compared with VEGF (positive control) with 100% HUVEC or HMVEC migration. Peptides were added in the presence of either 0.3 nM VEGF or 1 nM/5% FBS VEGF (bottom chamber). The data represent mean ± S.D. (*n*=3). Data were normalized to the mean VEGF control. *P*-values are represented as follows: ****P≤0.0001, ***P≤0.001, **P≤0.01 and *P≤0.05.

### Cell proliferation study on primary human endothelial and cancer cells

Although the cyclic TSP-1 mimetics showed significant inhibition in the endothelial cell migration assay, it was important to confirm that this activity was not an artefact of cell toxicity. Hence the toxicity of the peptides was screened against primary human cells (HUVECs and HMVECs) and selected cancer cell lines (HT-29, MCF-7 and PC-3). None of the peptides affected cell viability during a 2-h incubation (results not shown), which eliminates the possibility that peptide toxicity causes inhibition of cell migration. To evaluate the effect of these peptides on cells that were in a proliferative state, a longer incubation period was also tested. When the peptides were incubated with cells for up to 72 h, none of the peptides had an effect on HUVECs but a minor decrease in cell viability was observed on HMVECs, HT-29, MCF-7 and PC-3 cells, with IC_50_ values of more than 50 μM (Figures 4A and 4B), indicating that these peptides did not confer a strong reduction on cell viability in these cell lines.

**Figure 4:**
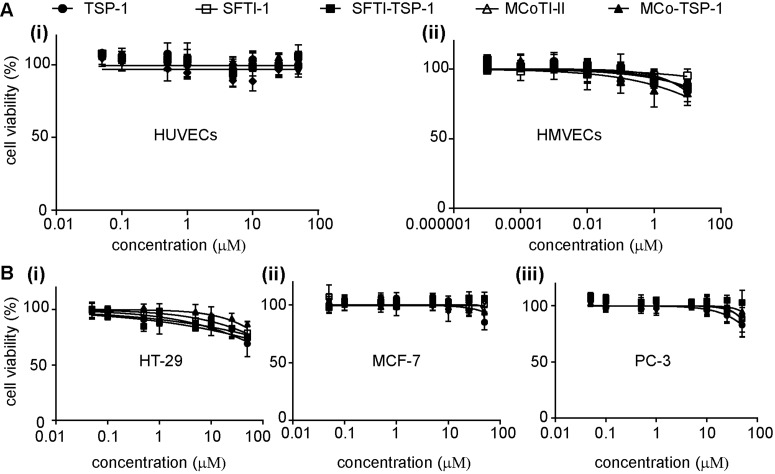
Cell proliferation assay All peptides were incubated for 72 h in five different cell lines, including the (**A**) primary human endothelial cells, (**i**) HUVECs and (**ii**) HMVECs, and (**B**) cancer cells, (**i**) HT-29, (**ii**) MCF7 and (**iii**) PC-3. Each peptide was tested in triplicate and the data represent mean ± S.D.

### Hemolytic activity and peptide stability

In addition to evaluating cytotoxicity and cell proliferation effects on human primary endothelial (non-cancerous) and cancer cells, it was also of interest to assess the toxicity of the peptides against human red blood cells. The peptides were tested in the concentration range of 0.01–50 μM and the native peptides and TSP-1 mimetics were non-haemolytic to human red blood cells when compared with melittin, a highly haemolytic peptide from honeybee venom (Figure 5A).

**Figure 5:**
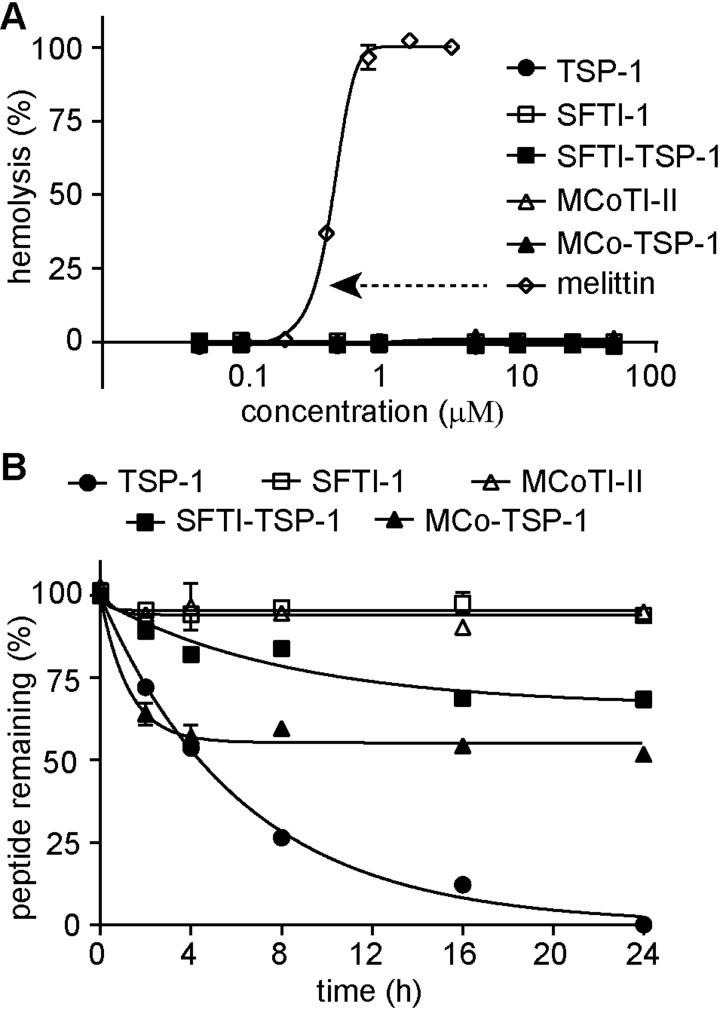
Evaluation of cyclic TSP-1 mimetics on human red blood cells and human serum (**A**) Hemolytic assays on peptides and frameworks, with melittin as a positive control. (**B**) Stability in human serum, with the graph showing the percentage of peptide remaining over a 24-h incubation. All data are represented as mean ± S.D. (*n*=3).

To assess the stability of the peptides, they were incubated in human serum. Linear TSP-1 heptapeptide was degraded within 24 h, in contrast with the cyclic peptides, as shown in Figure 5(B). The TSP-1 heptapeptide was degraded despite containing a D-amino acid. Although MCo-TSP-1 has lower stability than SFTI-TSP-1, more than 50% of both peptides remained in human serum over a 24-h period. Furthermore, both of these cyclic TSP-1 mimetics have enhanced stability compared with their linear counterparts.

## DISCUSSION

### Rational design of cyclic TSP-1 mimetics

The aim of the present study was to design cyclic thrombospondin-1 (TSP-1) mimetics using disulfide-rich frameworks and examine their effects on endothelial cell migration. Both MCoTI-II and SFTI-1 frameworks were used: the SFTI-1 framework contains a single disulfide bond that effectively divides the sequence into two backbone loops, termed the trypsin inhibitory loop (loop 1) and the secondary loop (loop 2); by contrast, the MCoTI-II framework contains three disulfide bonds and six loops. The linear heptapeptide (GViTRIR) was incorporated into loop 2 of SFTI-1 and loop 6 of MCoTI-II, with these loops chosen based on successful examples of the grafting of other epitopes into these loops [29–32,45]. The peptides were readily synthesized and folded and were highly soluble in water. Solubility is important, because a previous example of a TSP-1 derived peptide, ABT-526, led to challenges in pharmacokinetic studies in animals due to poor solubility [14]. The excellent solubility of the MCoTI-II and SFTI-1 frameworks favours their use in a wide range of grafting applications.

### Backbone cyclization approach improved stability and non-toxic to cells

We utilized non-natural amino acid substitution [46] and backbone cyclization [47] chemical approaches to improve linear TSP-1 fragment stability. Alternative approaches such as capping of the N- or C-termini [13] or N-methylation [48] have also been used to optimize bioactive peptides in other studies. The non-natural amino acid substitution strategy here involved replacing the first isoleucine residue of linear TSP-1 with D-Ile, as this modification is known to favour interaction with the CD36 receptor [13]. Another reason for this modification was the fact that D-amino acids can assist in improving stability, an important factor given that angiogenesis involves a vast range of proteolytic enzymes [49,50]. Both MCoTI-II and SFTI-1 contain a cyclic backbone that is amenable to incorporation of target epitopes, and this structural feature reinforced the stability of the TSP-1 mimetics; both cyclic TSP-1 peptides were more stable in serum over 24 h than the linear TSP-1 heptapeptide. SFTI-TSP-1 was more stable than MCo-TSP-1, probably due to the more rigid framework of SFTI-TSP-1. Importantly the NMR data showed that the linear TSP-1 fragment adopts a similar conformation in both frameworks. No toxic effects were observed for the peptides against human primary endothelial cells, cancer cells or red blood cells over a 2-h incubation period.

### Effects of linear, cyclic TSP-1 peptides and their native cyclic frameworks on cell proliferation study

Anti-proliferative activity was measured for all peptides over a 72-h period. None of the peptides showed any activity on HUVECs, probably due to the lack of CD36 expression in this cell line compared with HMVECs; however, the peptides had minor anti-proliferative effects on HT-29, MCF-7, PC-3 and HMVECs at high concentrations. The native (i.e. un-grafted) MCoTI-II and SFTI-1 native frameworks also had minor anti-proliferative effects on HT-29 [51] and PC-3, possibly due to the expression of matriptase receptors (type-II transmembrane serine protease) on these cells [52]. Although only a weak anti-proliferative effect was observed, Gray et al. [53] recently reported that MCoTI-II inhibited invasive cell migration in a matriptase over-expressed PC-3 cell line. It is possible that MCoTI-II has weak anti-proliferative activity by controlling growth of PC-3 cells but exhibits a stronger inhibitory effect on cell migration by controlling cell motility. The mild anti-proliferative effect coupled with the lack of toxicity observed in these peptides gives them an advantage over conventional cytotoxic drugs, which often have a narrow therapeutic index and high toxicity profile [54,55].

### Grafting of linear TSP-1 fragment into MCoTI-II and SFTI-1 affects endothelial cell migration

Figure 6 gives a schematic overview of the effects of the grafted cyclic peptides on cell migration. The linear and cyclic TSP-1 mimetics and native frameworks were examined in a human endothelial cell migration assay using two cell types, i.e., HUVECs and HMVECs. TSP-1 derived peptides target HMVECs through the CD36 receptor [12,56] and have an inhibitory effect on HUVEC migration via targeting the β-integrin receptor instead of CD36 [35]. We showed that cyclic TSP-1 mimetics have higher potency towards HMVECs compared with HUVECs. This difference in potency is likely a result of the difference in expression of CD36 receptors in HMVECs compared with large vessel cells such as HUVECs. Previous studies using flow cytometric analysis have shown the presence of CD36 on the surface of HMVECs, in contrast with HUVECs where the antibodies failed to detect the presence of CD36 [57,58]. This difference in potency is also consistent with previous studies on thrombospondin-1 type-1 repeats (TSR) by Short et al. [35], who reported 35% inhibition could be achieved on HMVECs at 5 nM but 16 nM was required for 60% inhibition on HUVECs. The TSP-1 heptapeptide grafted into the MCoTI-II framework resulted in better inhibition of cell migration than the SFTI-1 framework grafts, possibly because the larger framework provides better opportunity for the delivery of the TSP-1 fragment to its receptor than SFTI-1 [59]. Additionally, the site of the incorporation of the TSP-1 peptide in loop 6 of MCoTI-II offers greater flexibility compared with SFTI-1 as the SFTI-1 framework is small, with a β-hairpin constrained structure [26]. Although SFTI-TSP-1 was observed to be less potent than MCo-TSP-1, both peptides showed better stability and potency than their linear counterpart. On the other hand, the cell penetration properties of cyclic frameworks could also play a role in the different route of entry into cells, as D'Souza et al. [60] have recently shown that MCoTI-II is more efficiently internalized into cells than SFTI-1. This fits the results of cell migration study, where MCo-TSP-1 has better potency than SFTI-TSP-1. Although these peptides do not have as high potency as ABT-510 (inhibition of HMVEC migration: IC_50_=0.89 nM [14]), they are still active at low micromolar (SFTI-TSP-1) or nanomolar (MCo-TSP-1) concentrations. These data suggest TSP-1 mimetics act against HMVECs via a CD36-dependent pathway.

**Figure 6:**
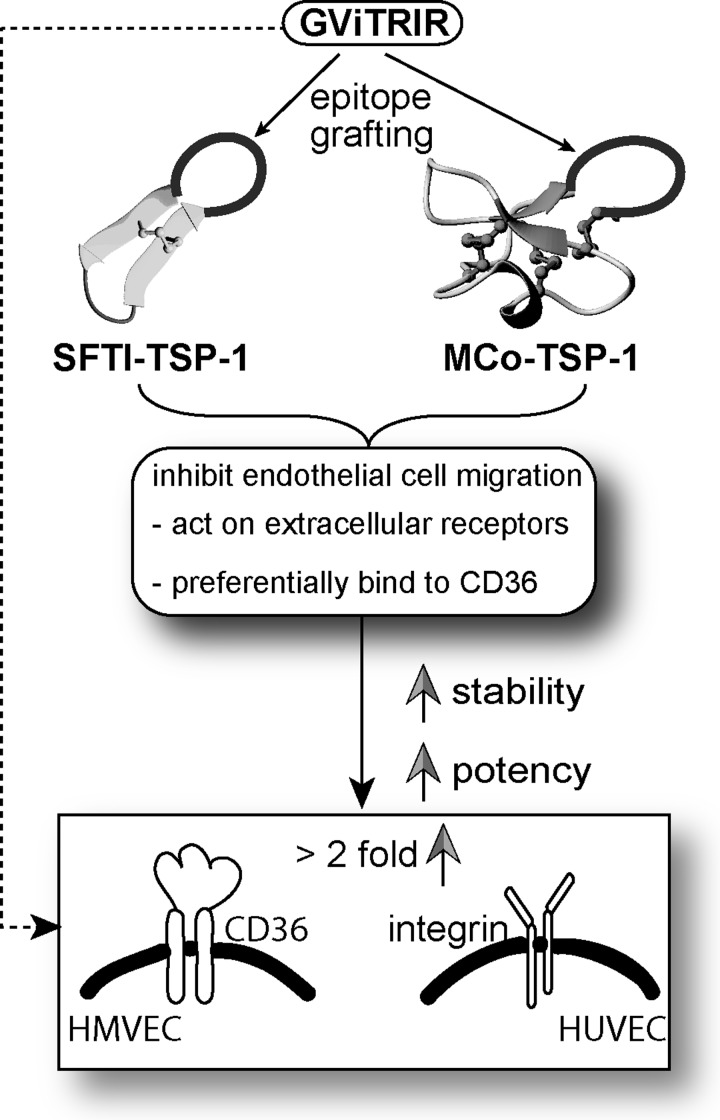
A schematic overview of the results from the present study Newly designed cyclic TSP-1 analogues (SFTI-TSP-1 and MCo-TSP-1) were able to suppress HMVEC migration. Interestingly, these peptides had better inhibition of HMVEC migration (highlighted with a square box) compared with HUVECs, probably due to the overexpression of CD36 receptors in HMVECs but not in HUVECs. It is also possible that these peptides have the ability to inhibit VEGF as well since VEGF was added during the migration assay. Weaker inhibition was also observed in HUVEC migration although CD36 was not expressed in these cells. This might be due to the possibility of TSP-1 being able to inhibit β1-integrin receptors expressed in HUVECs. This was also observed in Short et al. [35]. Overall, cyclic TSP-1 analogues have better stability and potency (pathway shown in solid line) than the linear heptapeptide TSP-1 (pathway shown in dashed line).

### Conclusions

Re-engineering linear TSP-1 fragment into cyclic frameworks appears to be a promising approach for developing potential anti-angiogenesis therapeutics. This approach resulted in improved stability and enhanced inhibition of endothelial cell migration without being toxic to human red blood cells, primary endothelial or cancer cells. Many FDA-approved cancer drugs (i.e. antibodies and small molecules) possess unwanted side effects, including increased risk of heart disease. The low toxicity profile of the cyclic TSP-1 peptides thus provides them a potential advantage over current drugs. Furthermore, since there are limited peptide-based drugs in the anti-angiogenesis field, the design and development of new generation drug candidates based on naturally occurring cyclic peptides provides an opportunity to better explore this therapeutic space. Although the cyclic TSP-1 peptides did not confer potency equivalent to ABT-510, they are active and exhibit excellent stability, thus demonstrating an opportunity for SFTI-1 and MCoTI-II frameworks in developing the next generation of thrombospondin mimetics. Further studies of these cyclic TSP-1 mimetics in animal tumour models will be important to evaluate the clinical relevance of these findings and to determine the interaction of these peptides to their corresponding receptors in *in vivo* settings. Overall, the present study has resulted in promising angiogenesis inhibitors with potential for therapy in cancer and other diseases caused by overgrowth of blood vessels.

## Abbreviations

DMEM: Dulbecco's modified Eagle's medium
EBM: endothelial basal media
HMVEC: human dermal microvascular endothelial cell
HUVEC: human umbilical vein endothelial cell
MCoTI-II: *Momordica cochinchinensis* trypsin inhibitor-II
NMR: nuclear magnetic resonance
RPMI: Roswell Park Memorial Institute medium
SFTI-1: sunflower trypsin inhibitor-1
TSP: thrombospondin
VEGF: vascular endothelial growth factor
